# Schadenfreude and the spread of political misfortune

**DOI:** 10.1371/journal.pone.0201754

**Published:** 2018-09-05

**Authors:** Laura C. Crysel, Gregory D. Webster

**Affiliations:** 1 Stetson University, Deland, FL, United States of America; 2 University of Florida, Gainesville, FL, United States of America; Technion Israel Institute of Technology, ISRAEL

## Abstract

Schadenfreude is a social emotion that describes one’s happiness at the misfortune of others. Because people experience schadenfreude to different extents, it can also be considered a trait. The present research aimed to develop a trait measure of schadenfreude and investigate the relationship between schadenfreude and political downfalls. We developed an item pool and used exploratory (Study 1) and confirmatory (Study 2) factor analyses to establish a 12-item, two-factor schadenfreude measure: benign and malicious. We also assessed its test–retest reliability (Study 3) and convergent validity with related measures (Study 4). Findings supported a two-factor schadenfreude measure that produced valid and reliable scores (Studies 1–4). In an experiment, we found a positive correlation between episodic—but not trait—schadenfreude on spreading news of a politician’s downfall (Study 5). Using a 3 (Political affiliation: Democrat, Republican, or other) × 3 (Manipulated condition: Democrat, Republican, or CEO) design, we examined the extent to which participants’ schadenfreude predicted their intentions and choices to share an embarrassing news story about a politician or CEO via social media. Schadenfreude can be assessed as a reliable trait—one that may help us predict why some people intend to spread news of embarrassing political failures.

## Introduction

*Schadenfreude* is pleasure at the misfortune of others [1]. It’s a social emotion distinct from envy (pain at the good fortune of others) and resentment (perceived injustice). Research has focused on distinguishing schadenfreude from other emotions [2] and determining the antecedents of schadenfreude [3]. Understanding schadenfreude is important because it can be central to everyday social relationships; people not only remember experiencing it (“He embarrassed himself, but it was *so* funny”), but also remember thinking that others were experiencing it at one’s expense (“They were glad that I failed”). Schadenfreude is an inter-group emotion that can extend to politics and nationality [1,3–7]. When people in one country celebrate the death of another country’s political leaders, they are experiencing schadenfreude. Given the increasing antagonism between U.S. political groups [8,9], schadenfreude is especially relevant to understanding reactions to news about opposing political parties.

For example, when a video surfaced of 2016 Republican Presidential candidate Donald Trump bragging about groping women without their permission, Republicans began to withdraw their support for him. One can imagine the schadenfreude of many Democrats when Trump’s campaign began to suffer. Similar to failing products [10], happiness at a politician’s misfortune may predict whether people share that misfortune with others via social media. This research expands on [10] to see if people’s failures are treated the same as products, and by connecting this episodic schadenfreude to trait differences in schadenfreude. In the present research, we provide an overview of the prior research on schadenfreude, develop a new measure of it, and investigate its role in spreading news of misfortune that befalls someone from an opposing political party.

### Episodic schadenfreude

Barring one exception [11], schadenfreude has been measured as a response to a vignette in prior studies, in which researchers ask participants to express how happy they feel regarding a specific event. More positive responses are considered higher in schadenfreude, though it is more clearly labeled as episodic schadenfreude. While research has shown differences in *episodic* schadenfreude, there is little research on individual differences in *trait* schadenfreude. Although schadenfreude is a state-like emotion, people also vary in their expression of schadenfreude or their reaction to schadenfreude-producing situations.

People vary in the extent to which they enjoy another’s misfortunes. There are two primary reasons for variation in trait schadenfreude. First, there is a similar distinction between trait- and state-level envy, which is a similar-but-distinct emotion that also exhibits trait-level individual differences [12]. If people differ in envy, then they may also differ in schadenfreude. Second, one study has already attempted to measure trait-level schadenfreude [11], and found that it related to dispositional envy.

In a study on envy and its relationship to narcissistic vulnerability [11], researcher developed an ad-hoc six-item trait-level schadenfreude scale. Despite that no formal psychometric testing was done in developing this schadenfreude scale—in part because the article focused on narcissism—it had good reliability (α = .90) and principal components analysis revealed a single factor. Although two of the items ostensibly measured schadenfreude (“Seeing others fail can sometimes feel good” and “When someone I dislike fails at something, it makes me smile”), one appeared to measure resentment (“When somebody beats me at something, I secretly hope something bad will happen to them”), and three appeared to assess envy (“I take pleasure in somebody better than me having a setback,” “Hollywood celebrities get what is coming to them,” and “Somebody that has what I want should ‘not have it all”). As we discuss below, both envy and resentment are distinct from schadenfreude [2, 13,14]. Items that more directly target schadenfreude may provide better construct validity. The goal of prior research was to measure the connections among trait-level schadenfreude, envy, and narcissism [11]. Consequently, this initial schadenfreude scale served its purpose, but its validity remains unestablished. In the present work, we sought to develop a new measure that focuses exclusively on trait schadenfreude (vs. envy or resentment).

### Schadenfreude, politics, and communication

One goal of the present research was to examine the relationship between schadenfreude and political downfalls. Specifically, schadenfreude involves more than simply conveying one’s pleasure at a misfortune; it may also consequently increase the likelihood that one will share the news of that failure. For example, schadenfreude was associated with intention to communicate negative information about a failed product [10]. Schadenfreude—in this case happiness at the product’s failure—correlated .28 (*p* < .001) with intentions to tell others about an envied car’s breakdown. People experiencing schadenfreude were also more likely to change their minds about the brand and feel more negatively about it. Similarly, participants experiencing malicious envy felt colder toward the car’s owner, hoped the owner would fail, and complained to another person about the owner [15]. Thus, products invoking envy via advertisement may be wielding a double-edged sword if the product fails [10]. Nevertheless, it could be that schadenfreude predicts the spread of news even when envy isn’t involved.

Because of its polarized two-party system, schadenfreude may be especially relevant to U.S. politics. For example, negative events for an opposing political party’s candidate led to increased schadenfreude for the other group [4]. Schadenfreude occurred even when negative events for the opposing party affected innocent people. Negative events such as a stagnant economy and troop casualties produced relatively higher schadenfreude when it would also be politically damaging for the other party. The current research investigates people’s willingness to spread news of such negative events to others.

### Overview

The present research had two main goals: (1) develop a measure of trait schadenfreude and (2) assess the link between schadenfreude and spreading news of politicians’ failures. To achieve these goals, we conducted five studies with adequate statistical power (≥ .80) to detect either (a) the mean effect size in social-personality psychology (i.e., *r* = .21; [16]) or (b) in the special case of our test–retest reliability study (Study 3), a sample size large enough to detect a typical test–retest correlation (i.e., *r* = .70). First, we evaluated a pool of 54 potential schadenfreude items and distilled it into a 12-item scale (Study 1). Second, using confirmatory factor analysis, we examined the structure of our 12-item schadenfreude scale (Study 2). Third, we assessed our schadenfreude scale’s test–retest reliability (Study 3) and construct validity by examining its place within a nomological network of related traits (Study 4). Finally, we also sought to replicate and extend the relationship between schadenfreude and intentions to spread word of a product’s failure [10] to the political domain (Study 5). Specifically, Study 5 used a fictional news story about a person that was either a politician (Democrat or Republican) or a CEO (non-partisan control). Participants reported schadenfreude for this target individual’s downfall and reported their intention to share the news story via social media. We included a behavioral measure: participants chose whether to share the story on Facebook or Twitter.

## Study 1: Developing schadenfreude scale items and exploring its factor structure

In Study 1, our goals were to generate a pool of potential items for our schadenfreude scale and perform factor analyses to cull the item pool and evaluate the structure of our measure.

### Generating initial schadenfreude item pool

Nine undergraduate research assistants listened to a short video about schadenfreude, which defined it as “people taking pleasure in your pain,” and were given examples of schadenfreude (e.g., laughing at a clumsy athlete). They then generated items that could be used to measure someone’s experience of schadenfreude. Correcting items for grammar, clarity, and specificity resulted in a pool of 54 schadenfreude items; six were reverse-coded.

### Method

#### Participants

Participants were 802 people (395 men, 389 women, 18 unspecificed; ages: 18–71 years, *M* = 28.6, *SD* = 11.3), with 212 (26%) Introductory Psychology students and 590 (74%) members of Amazon’s Mechanical Turk (MTurk; http://mturk.com). MTurk participants are typically no more biased or inattentive than undergraduate samples [17,18]. We compensated Mturk participants with US11¢ and psychology students with one credit toward a research participation requirement. The Behavioral/NonMedical IRB (IRB02) of the University of Florida approved this research.

#### Materials and procedure

Using a response scale from 1 (s*trongly disagree*) to 9 (s*trongly agree*), participants rated their agreement with the 54 potential schadenfreude items, as well as the abovementioned six-item schadenfreude scale [11].

### Results

**Fig 1:**
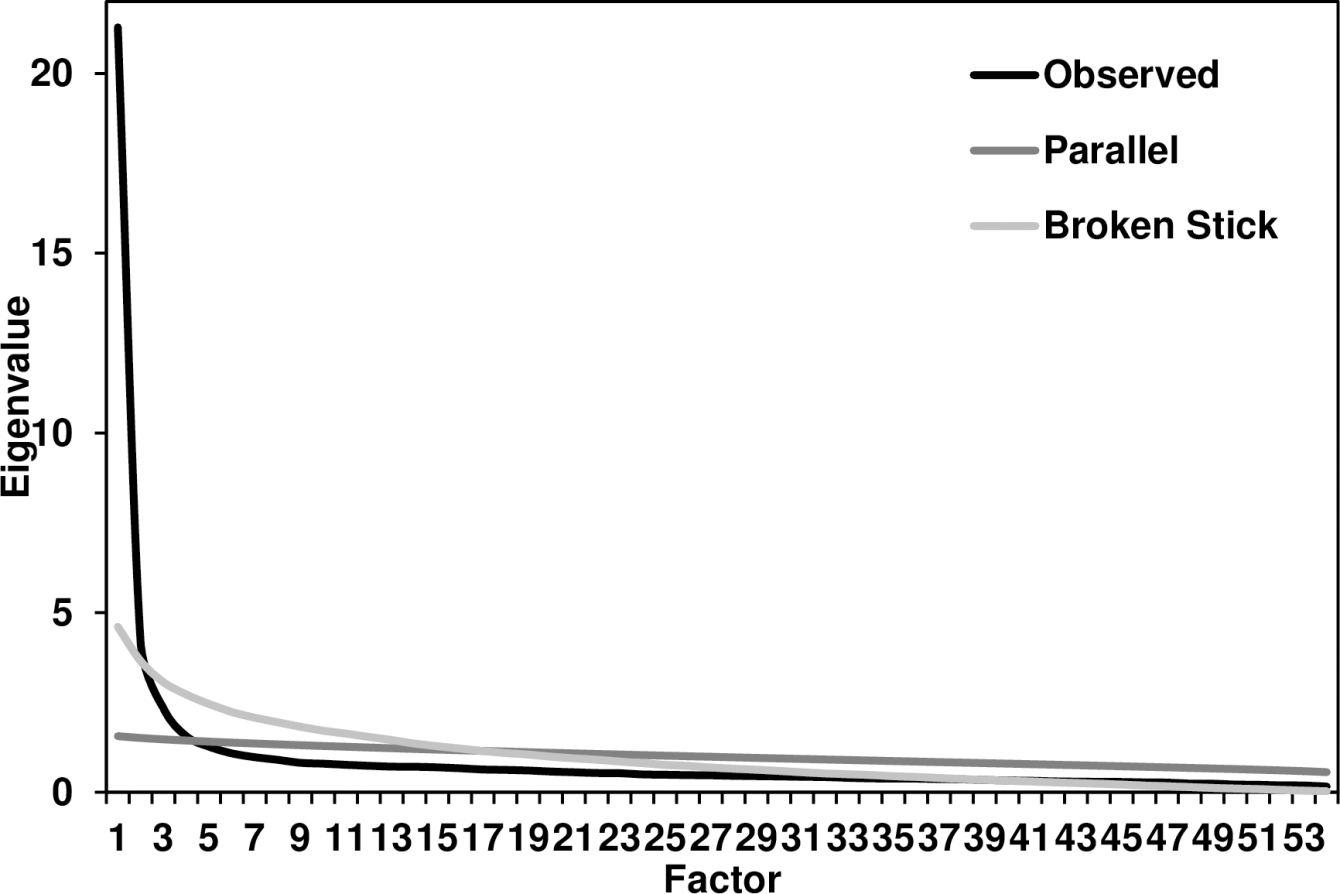
Factors in Study 1. Scree plots of Eigenvalue by number of factors: Observed, parallel analysis, and broken-stick analysis.

**Fig 2:**
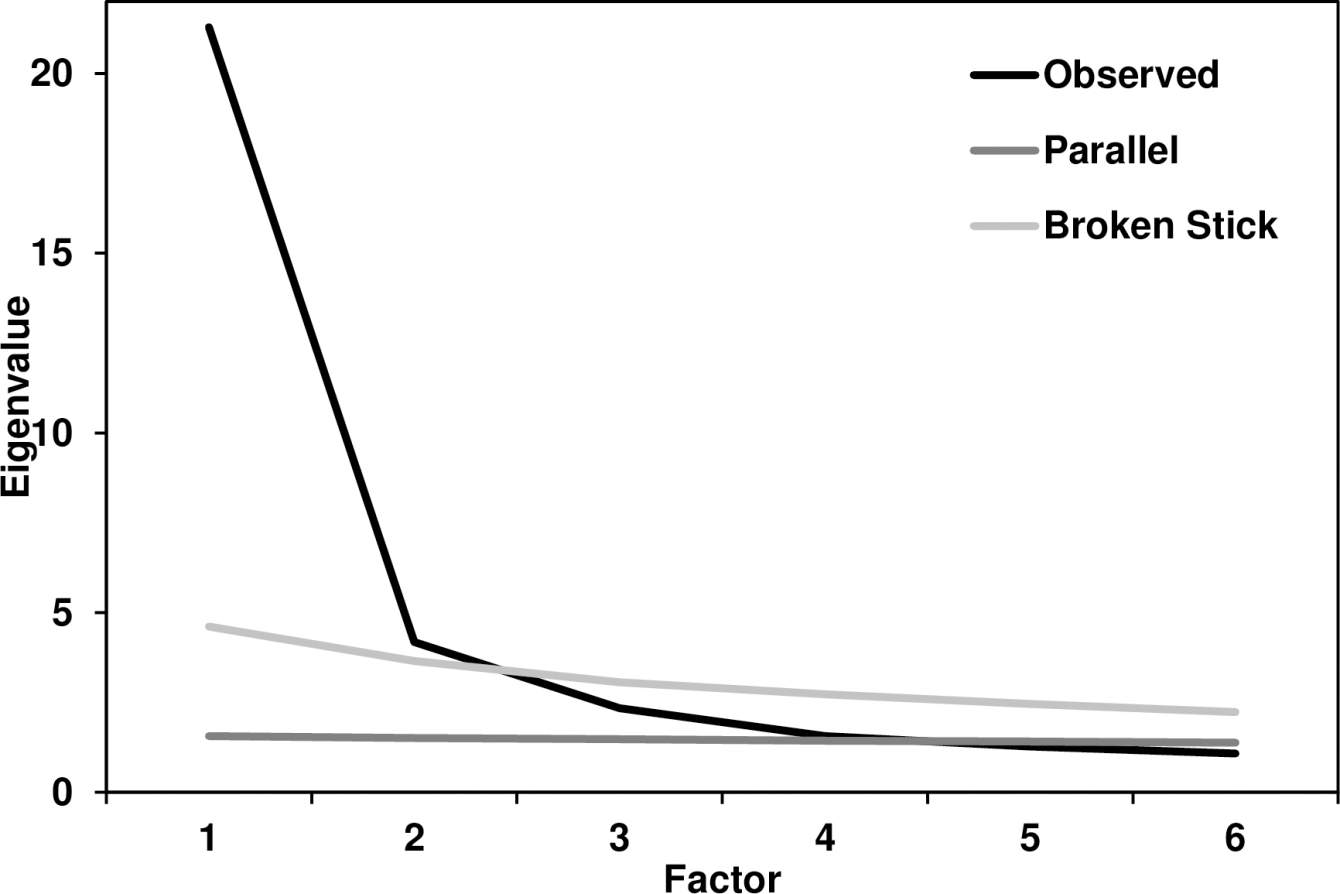
Close-up of Fig 1. Close-up of Fig 1 showing scree plots of Eigenvalue by the first six factors: Observed, parallel analysis, and broken-stick analysis.

We used principal axis factor (PAF) analyses with oblique rotation on the 54-item schadenfreude scale. We chose PAF over other factor-analytic methods (or principal components analysis) because PAF is preferred when the analytic goal is to detect latent structure. Six components had Eigenvalues ≥ 1.0 and together accounted for 58.72% of the variance, but the scree plot suggested a three-factor solution (Figs 1 and 2). A parallel analysis [19] suggested retaining four factors, whereas a broken-stick analysis [20] suggested retaining two; we chose to split the difference and retained three factors for subsequent analysis. We labeled the first two factors as “benign” schadenfreude (in which little harm occurred) and “malicious” schadenfreude (in harm was present); a third “method” factor consisted of the six reverse-scored items. For example, benign schadenfreude is akin to laughing at a video of a cat falling off a counter, whereas malicious schadenfreude is closer to the joy some people experience when others lose their jobs [4,10]. We then excluded the six reverse-scored items from our initial scale because they confounded method variance with substantive variance (they loaded on neither the benign nor malicious factors); we revisit this issue in Study 2.

The next PAF analysis retained the benign and malicious schadenfreude factors for a two-factor solution. Next, we chose the items with the highest loadings (Table 1), while removing redundant items. We removed some items for face validity because they appeared to have more to do with sadism (“I like to be the cause of someone’s pain”). Finally, we cut some items because they were culture-specific (e.g., *American Idol*). The result was a 12-item scale with good reliability (α = .89, *M* = 3.65, *SD* = 1.49) and a clear two-factor structure. The benign (α = .84, *M* = 4.63, *SD* = 1.82) and malicious (α = .91, *M* = 2.67, *SD* = 1.63) subscales also had good reliability.

**Table 1:** Factor loadings for schadenfreude items.

	54-item (PAF)			48-item (PAF)		12-item (CFA)	
Item	1	2	3	1	2	1	2
24.I like to be the cause of someone’s pain.	.91	-.16	.05	.94	-.20		
28.I take pleasure in being the cause of someone else’s pain.	.90	-.15	.03	.92	-.18		
23.I like to be the cause of someone’s ruin.	.89	-.17	.04	.90	-.21		
19.I like to watch people cry.	.87	-.11	.02	.89	-.14		
25.I like to watch people in pain.	.86	-.06	.06	.90	-.10		
**26.I take pleasure in another’s failure.**	.86	-.03	-.02	.86	-.03	.85	-.01
**12.I enjoy seeing someone’s computer crash.**	.81	-.04	.05	.84	-.07	.81	-.02
**10.I enjoy it when others get low grades.**	.80	.01	-.08	.78	.03	.75	.04
**17.I like to see someone successful get fired.**	.80	-.05	.00	.81	-.06	.81	-.06
**11.I like watching others on their bad day.**	.80	.02	.07	.83	-.02	.82	-.01
3.I secretly feel happy when a friend gets a poor grade on an exam.	.77	.00	-.08	.75	.02		
33.I feel satisfied when classmates fail a test.	.73	.06	-.02	.73	.07		
49.I enjoy watching other people fail.	.73	.12	.01	.74	.10		
35.I feel good when I see people walking in the rain while I’m driving in my car.	.71	.00	.05	.73	-.03		
9.I have trouble being happy for other people’s achievements.	.70	-.06	-.14	.67	-.02		
**40.I laugh when someone just misses the bus.**	.68	.15	.09	.71	.11	.70	.13
29.Seeing or hearing about another’s misfortune makes me feel better about myself.	.64	.11	-.06	.62	.06		
38.I feel superior when a peer’s joke doesn’t go over well.	.64	.11	-.02	.63	.11		
44.I think it’s funny when I see someone get rejected.	.62	.23	.02	.63	.22		
53.I sometimes laugh when I see a child crying.	.61	.08	.01	.62	.06		
34.I’m amused when someone who raises his or her hand gets a question wrong.	.57	.19	-.03	.57	.19		
18.I like to watch people become irritated.	.55	.30	.08	.58	.26		
36.I feel good when the car behind me misses the light.	.50	.25	.02	.50	.25		
13.I feel better about myself when others are unprepared for class.	.49	.22	-.14	.44	.26		
16.I think it’s funny when other people are embarrassed.	.45	.40	.14	.49	.35		
27.I take pleasure in someone tripping.	.44	.34	.11	.47	.30		
51.I find it amusing when random couples argue.	.44	.30	-.02	.43	.30		
39.I feel more competent when another makes a mistake.	.44	.24	-.19	.38	.31		
21.I like to see one of my teachers make mistakes.	.43	.31	.03	.43	.31		
32.When I watch movies in which everything seems to go wrong for the main character(s), I find them hilarious.	.35	.32	.12	.39	.27		
**37.It’s funny when a person walks into a closed sliding-glass door.**	-.11	.78	-.11	-.15	.82	-.13	.81
50.I sometimes laugh at clumsy people.	-.03	.74	-.06	-.05	.76		
**1.I enjoy watching segments of videos where people fall.**	.03	.73	-.01	.03	.72	.02	.77
**8.I enjoy reading “most embarrassing moment” stories.**	-.05	.69	-.03	-.06	.70	-.04	.70
**6.I have laughed at someone who has fallen before helping them up.**	.00	.65	-.06	-.01	.67	.01	.65
5.I like watching footage of terrible auditions (for example, American Idol).	-.08	.63	-.05	-.09	.64		
45.I think it’s funny when drunk people spill drinks on themselves in bars.	.07	.57	-.09	.04	.60		
**54.I think it’s funny when I see a person make a fool of himself or herself.**	.23	.56	.09	.25	.53	.27	.49
**4.I enjoy slapstick comedy where characters get hurt.**	.13	.54	.07	.15	.50	.17	.52
48.I think it’s funny when frat guys get turned down.	.07	.54	-.18	.02	.59		
14.I enjoy watching videos where performers may get hurt.	.37	.47	.13	.41	.42		
7.I have wanted to laugh at a friend’s unfortunate story.	.29	.46	.00	.29	.46		
52.I enjoy watching people fight (either on TV or in real life).	.38	.42	.13	.41	.38		
20.I like to see someone trip.	.35	.42	.11	.38	.38		
46.I laugh when I see people who are driving hit curbs/mailboxes.	.39	.39	.06	.41	.36		
22.I like to watch horror films.	.01	.38	.06	.02	.36		
41.It makes me happy when I see that my ex has gotten fat or ugly (for example, on Facebook).	.19	.38	-.10	.15	.42		
42.It makes me happy when people that used to be popular in high school amount to nothing special.	.32	.37	-.14	.27	.43		
2.I feel embarrassed for other people when they embarrass themselves.†	.08	-.17	.60				
15.I feel sorry for people when they are reprimanded.†	.00	-.05	.59				
43.I feel bad when I see someone in public with a rip in the back of their pants.†	.01	-.08	.53				
31.Movies in which everything seems to go wrong for the main character(s) make me uncomfortable.†	-.19	.18	.47				
30.When something bad happens to someone else, I feel sad.†	.21	-.09	.46				
47.I feel bad when celebrities who were drug addicts die.†	-.02	-.01	.42				

Note. PAF = Principal Axis Factoring; pattern matrix show. CFA = Confirmatory Factor Analysis. *N* = 754 after listwise deletion.

†Reverse-scored item.

We ran confirmatory factor analyses (CFAs) on the retained 12 schadenfreude items—six from each factor or subscale. We assessed goodness-of-fit using the following measures (and established criteria; [21]): Chi-squared test (χ^2^; *p* > .05), Standardized Root Mean Square Residual (SRMR; ≤ .08), Root Mean Square Error of Approximation (RMSEA; ≤ .08), Comparative Fit Index (CFI; ≥ .90), and Tucker-Lewis Index (TLI; ≥ .90). The one-factor model fit the data poorly (χ^2^_54_ = 1,058, *p* < .001; CFI = .78; TLI = .74; SRMR = .11; RMSEA = .16, 90% CI [.15, .17], *p* < .001), but the two-factor model fit the data well (χ^2^_53_ = 220, *p* < .001; CFI = .96, TLI = .96, SRMR = .052; RMSEA = .065, 90% CI [.056, .074], *p* = .003). The two-factor model fit the data better than the one-factor model (Δχ^2^_1_ = 838, *p* < .001). All items loaded onto their predicted factors. The two factors were correlated (*r* = .56, *p* < .001), suggesting that they overlapped, but still measured distinct aspects of schadenfreude. Showing convergent validity, the six-item Schadenfeude measure [11] correlated positively with our new 12-item scale (*r* = .70) and its malicious (*r* = .75,) and benign (*r* = .54) subscales (*p*s < .001). We caution readers that testing exploratory (PAFs) and confirmatory (CFAs) models using the same data can be problematic because of non-independence. Thus, we again tested CFAs using new data in Study 2.

### Discussion

Study 1 produced a 12-item, two-factor trait measure of schadenfreude with reasonable fit. Because of the items loading on each factor, we labeled them as benign schadenfreude and malicious schadenfreude. We jettisoned six reserve-coded items from the original 54-item pool because they confounded valence with substantive construct dimensions. Negative items are important in scale development because they can reduce positive response bias [22]. In Study 2, we address this issue altering six of the 12 items to be reverse-coded by adding the word “not.” We then ran additional CFAs to assess the fit of our updated 12-item schadenfreude measure.

## Study 2: Confirming factor structure

In Study 2, we reverse-coded six of our 12 retained schadenfreude items to make our new scale less prone to positive response bias and socially desirable responding. Reducing these biases is important for schadenfreude because people often disagree more with socially undesirable items, and negatively-valenced items can help reduce this bias [22]. For example, people may find it *easier* to agree *less* with the statement “I do not take pleasure in another’s failure” than to agree *more* with the statement “I enjoy reading ‘most embarrassing moment’ stories.” Nevertheless, there are some disadvantages to altering these items, such as potential factor-structure changes. Rather than simply confirming the factor structure from Study 1, one may need to account for differential responding to reverse-scored items using a “method factor.” We expected that the two-factor structure would persist after controlling for method variance with an additional factor for negatively-valenced items.

### Method

#### Participants

Mturk participants (*N* = 212; 101 men, 103 women, 8 unspecificed; ages: 18–68 years, *M* = 33.0, *SD* = 12.3) completed our 12-item schadenfreude measure; they were compensated US11¢. Fourteen participants were removed for failing an attention check item (i.e., “Please select ‘agree’ for this item”), and six were removed for failing to answer all of the items. Analyses were based on the remaining 192 participants.

#### Materials and procedure

Participants provided demographic information and rated their agreement from 1 (s*trongly disagree*) to 9 (*strongly agree*) with our updated 12-item schadenfreude scale, which included six reverse-scored items to reduce response bias (Table 2).

**Table 2:** Means and standard deviations for the 12-item schadenfreude scale.

Item	Mean	*SD*
1. I do not enjoy watching segments of videos where people fall.	5.12	2.36
2. I do not enjoy slapstick comedy where characters get hurt.	5.21	5.35
3. I have laughed at someone who has fallen before helping them up.	5.68	2.26
4. I enjoy reading “most embarrassing moment” stories.	5.85	2.34
5. It’s not funny when a person walks into a closed sliding-glass door.	5.54	2.57
6. I think it’s funny when I see a person make a fool of himself or herself.	5.07	2.19
7. I enjoy it when others get low grades.	2.62	1.83
8. I do not like watching others on their bad day.	3.21	2.00
9. I do not enjoy seeing someone’s computer crash.	2.91	2.13
10. I like to see someone successful get fired.	2.53	1.88
11. I do not take pleasure in another’s failure.	3.31	1.98
12. I laugh when someone just misses the bus.	2.92	2.02

Note. The first six items are the “benign” items and items seven through twelve are the “malicious” items. The response scale was from 1 (strongly disagree) to 9 (strongly agree). Negatively valenced items were reverse-scored.

### Results

**Fig 3:**
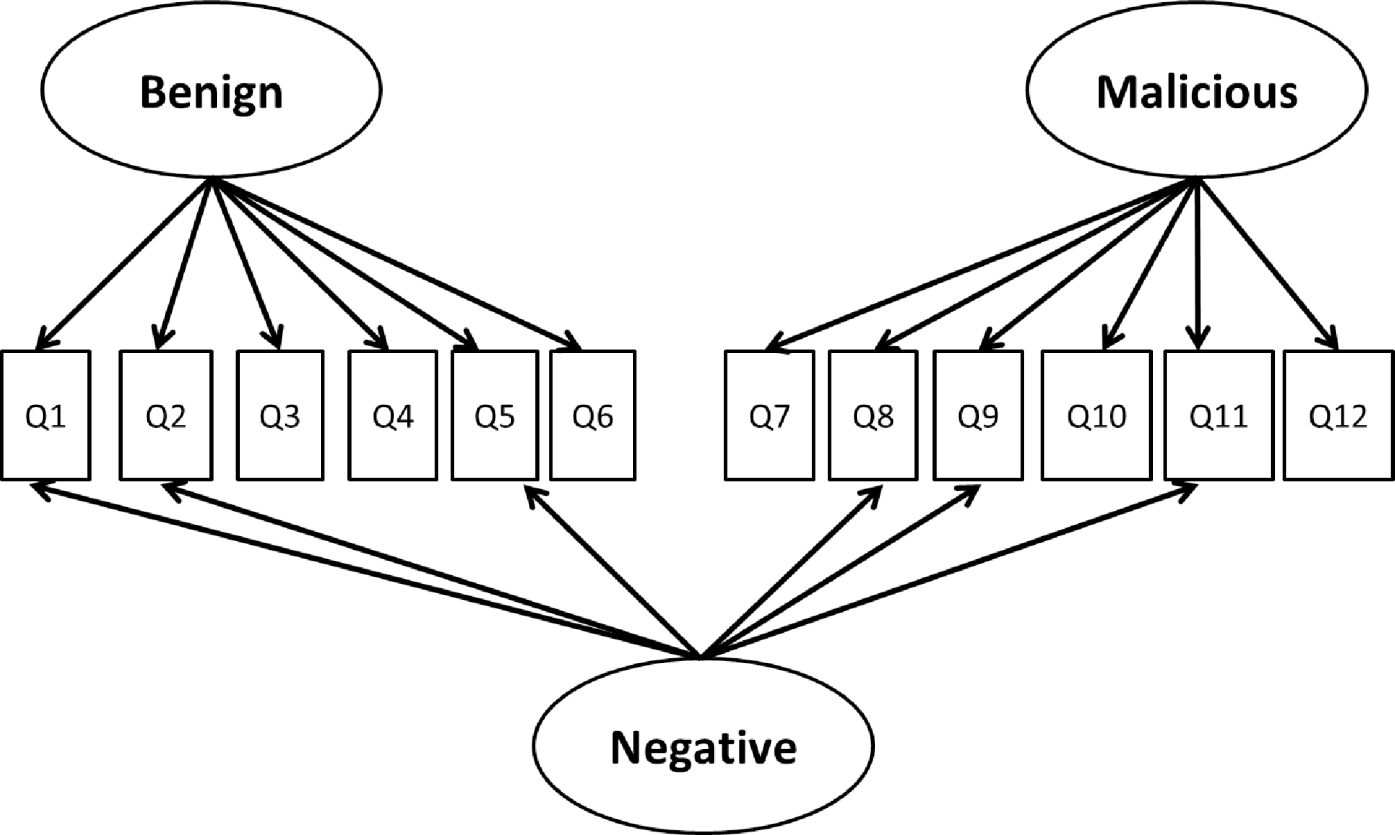
Final factor structure of the schadenfreude measure. The benign and malicious factors were moderately correlated (*r* = .58, *p* < .001). The negative valence factor was uncorrelated with the benign (*r* = -.08) and malicious (*r* = -.43) factors (*p*s > .06).

Using CFAs, we again expected the two-factor model to fit the schadenfreude item data better than a single-factor one. The one-factor schadenfreude model fit the data poorly (χ^2^_54_ = 277, *p* < .001; CFI = .69; TLI = .62; SRMR = .11; RMSEA = .15, 90% CI [.13, .16], *p* = .001). Although the two-factor model fit the data better than the one-factor model (Δχ^2^_1_ = 127, *p* < .001), the fit for the two-factor model was relatively poor (χ^2^_53_ = 150, *p* < .001; CFI = .86; TLI = .83; SRMR = .075; RMSEA = .098, 90% CI [.079, .12], *p* < .001). The best fit to the data resulted from adding a third “method factor” to the model to account for the negative valence of six reverse-scored items (Fig 3). Fit was acceptable (χ^2^_45_ = 88, *p* < .001; CFI = .94; TLI = .91; SRMR = .053; RMSEA = .070, 90% CI [.048, .092], *p* = .063) and showed significant improvement over the two-factor model (Δχ^2^_8_ = 62, *p* < .001). The benign and malicious factors were correlated (*r* = .58, *p* < .001). The negative valence factor was uncorrelated with the benign (*r* = -.08) and malicious (*r* = -.43) factors (*p*s > .06). All items loaded onto their predicted factors significantly but one (“I do not enjoy seeing someone’s computer crash”), which failed to load onto the reverse-scored factor.

### Discussion

We expected our 12-item schadenfreude measure would consist of two factors, benign schadenfreude and malicious schadenfreude. After controlling for negatively-valenced items with a method factor, the resulting CFA supported the expected two-factor structure for the 12 schadenfreude items, which yielded an acceptable fit to the data.

Although model fit improved after including a method factor, this does not necessitate special scoring for the six reverse-scored items. Indeed, researchers can and should score each subscale—benign and malicious—by first reverse-scoring the negatively-valenced items and then averaging across items within each subscale. The resulting composite subscales should produce valid and reliably scores for benign and malicious schadenfreude, as shown in Studies 3–5 below. This is also the case with one of the most popular measures in social science—Rosenberg’s Self-Esteem Scale (RSES; [23])—featuring 10 items, 5 negatively-valenced. Again, despite that CFA model fit improves when including a method factor, the RSES continues to be scored as a composite by taking a simple mean across items following reverse-scoring [24].

In sum, although Studies 1 and 2 confirmed our schadenfreude scale’s two-factor structure, questions remained regarding its test–retest reliability (Study 3), convergent validity (Study 4), and predictive validity in a controlled experiment (Study 5).

## Study 3: Test–retest reliability

We next sought to assess our schadenfreude scale’s test–retest reliability because trait-based individual differences should be stable over time. We expected that our schadenfreude scale would show adequate rest–retest reliability (*r*s ≥ .70).

### Method

Undergraduates in a social psychology course completed our 12-item schadenfreude measure twice, four weeks apart. Schadenfreude was measured using the same items and response scale described in Study 2. There were originally 45 participants, but only 28 (62%) completed items at both time points; all analyses were run on these 28 participants (age and gender information were not collected).

### Results

Time 1 reliability was lower than expected but still acceptable for the total scale (*α =* .62, *M* = 4.03, *SD* = 0.91), the benign subscale (*α* = .62, *M* = 5.79, *SD* = 1.28), and the malicious subscale (*α* = .51, *M* = 2.26, *SD* = 1.05). However, reliability improved at Time 2 for the total scale (*α* = .76, *M* = 3.94, *SD* = 1.05), the benign subscale (*α* = .68, *M* = 5.48, *SD* = 1.31), and the malicious subscale (α = .77, *M =* 2.40, *SD* = 1.29). Importantly, the test–retest correlation for the total scale was high (*r* = .87), as was the correlation for the benign (*r* = .73) and malicious (*r* = .84) subscales (*p*s < .001).

### Discussion

Overall, results supported the test–retest reliability of our 12-item schadenfreude scale. Adequate test–retest reliability suggests that people were relatively stable in trait schadenfreude. Reliability was lower in Study 3 than in Studies 1–2; however, this may relate to Study 3’s small sample (*N* = 28) because smaller samples have greater standard errors. Although the findings of Studies 1–3 establish the 12-item schadenfreude scale’s factor structure and test–retest reliability, they cannot speak to its convergent validity. Thus, Study 4 examined whether our 12-item trait schadenfreude scale correlated with relevant traits.

## Study 4: Convergent validity

In Study 4, we sought to establish convergent validity between our new schadenfreude scale and several related measures, including an earlier six-item schadenfreude measure [11], envy, empathy, humor, aggressive humor, aggression, the Dark Triad (i.e., narcissism, psychopathy, and Machiavellianism), the Big Five personality traits, social desirability, self-esteem, and gratitude.

### Method

#### Participants

Participants were 350 Mturk members, who were compensated US26¢. We included three attention-check questions throughout the survey, and removed participants who failed one or more of these. Consequently, 261 participants (75%) were retained for analysis (144 men, 115 women, 2 unspecified; ages: 18–68 years, *M =* 31.2, *SD =* 10.1).

#### Materials and procedure

Participants completed the same 12-item schadenfreude measure and response scale used in Studies 2–3. They also completed several other measures (described below) that relate to schadenfreude.

#### Alternative schadenfreude scale

Participants completed Krizan and Johar’s six-item schadenfreude measure [11] using a 1 (s*trongly disagree*) to 7 (*strongly agree*) response scale. As noted above, this earlier scale had good internal consistency, but contained only two clearly face-valid schadenfreude items and was developed without any psychometrically rigorous testing (that was not the original study’s goal). We expected that our trait-level schadenfreude scale would correlate positively with this alternative six-item schadenfreude measure.

#### Envy

Envy—a negative emotional state brought about by upward social comparison—was measured using the eight-item Dispositional Envy Scale ([12]; e.g., “I feel envy every day.”). Research on schadenfreude and envy has yielded inconsistent results: most studies find either null or positive correlations. It may be that when a person is envious of a similar other, and therefore relevant for self-evaluation, the envy–schadenfreude link is stronger [25]. Participants rated their agreement from 1 (*strongly disagree*) to 5 (*strongly agree*). Because Krizan and Johar’s schadenfreude [11] measure contains items closely related to envy, we expect their measure to correlate more positively with dispositional envy than ours.

#### Empathy

We assessed empathy using the Toronto Empathy Questionnaire, a 16-item measure of emotional and cognitive responses to another’s emotions [26]; e.g., “I find that I am ‘in tune’ with other people’s moods”). Because research has addressed empathy as both emotional (compassion) and cognitive (perspective-taking), this empathy scale consisted of items from multiple earlier empathy measures to achieve a broad, single-factor measure. Participants rated their agreement from 1 (n*ever*) to 5 (a*lways*). We expect schadenfreude to correlate negatively with empathy ([4]).

#### Humor

We measured humor using the six-item Sense of Humor Questionnaire, which examines individual differences in amusement tendencies ([27]; e.g., “Would it be easy for you to find something comical, witty or humorous in most situations?”). Participants rated their agreement from 1 (*Very Easy*, *Not at all*, *Very much*) to 4 (*Very difficult*, *Yes indeed*, *Very little*). Because of the comparatively negative nature of our scale’s items, humor should correlate negatively with schadenfreude.

#### Aggressive humor

We measured aggressive humor using the eight-item Aggressive Humor Subscale of the 32-item Humor Styles Questionnaire ([28]; e.g., “If someone makes a mistake, I will often tease them about it”). Aggressive Humor style is hostile and enhances the self at the expense of others. Other styles include enhancing the self at no one else’s expense (Self-Enhancing), enhancing relationships (Affiliative), and enhancing relationships at one’s own expense (Self-Defeating). Participants rated their agreement from 1 (*totally disagree*) to 7 (t*otally agree*). Because of its similarity to schadenfreude, we expected a substantial positive correlation.

#### Dark triad

The Dark Triad is a set of three positively interrelated subclinical traits: Machiavellianism, psychopathy, and narcissism [29]. These traits often correlate with risk-taking and exploitative strategies [30, 31]. We measured these traits using the Dark Triad Dirty Dozen (DTDD; [32,33]), a 12-item scale that provides reliable scores. Machiavellianism is characterized by a sense that “the ends justify the means.” Psychopathy is associated with callousness and lacking empathy. Narcissism is obsessive or excessive self-love. Items include “I tend to exploit others towards my own end” (Machiavellianism), “I tend to lack remorse” (psychopathy), and “I tend to seek prestige or status” (narcissism). Participants rated their agreement from 1 (*strongly disagree*) to 9 (s*trongly agree*).

#### Aggression

We measured trait aggression using the Brief Aggression Questionnaire (BAQ; [34,35]). The BAQ is a 12-item measure of individual differences in trait aggression consisting of 4 three-item subscales: physical aggression, verbal aggression, anger, and hostility. Participants rated their agreement from 1 (s*trongly disagree*) to 7 (*strongly agree*). Items include “Given enough provocation, I may hit another person” (physical aggression); “My friends say that I’m somewhat argumentative” (verbal aggression); “I have trouble controlling my temper” (anger); and “Other people always seem to get the breaks” (hostility). Because prior research has not studied schadenfreude with specific types of aggression, we were agnostic regarding how these scales would relate.

#### Personality

The Mini International Personality Item Pool (Mini IPIP) is a 20-item measure includes five subscales, each of which assesses one of the Big Five personality traits: extraversion, agreeableness, conscientiousness, neuroticism, and openness [36]. The Mini IPIP was developed to provide efficient measures of the Big Five traits, and it shows similar test–retest and convergent validity correlations to those of longer Big Five scales. Participants rated their agreement from 1 (*very inaccurate*) to 5 (*very accurate*). Although schadenfreude has not been examined a Big Five context, we expected that it would relate positively to neuroticism and negatively to both agreeableness and conscientiousness; we remained agnostic about its relation to openness and extraversion.

#### Social desirability

We measured social desirability with the Social Desirability Scale Short Form C, a 13-item scale that examines bias in people’s responses to socially acceptable responses ([37–39]; e.g., “I have never deliberately said something that hurt someone’s feelings.”). People may be biased in their self-reports to the extent that they report the answers that they believe will gain others’ approval. Participants rated their agreement on dichotomous, true–false response scales. By definition, schadenfreude is a positive response to another’s misfortune, and should thus correlate negatively with social desirability. People who report low schadenfreude may be uncomfortable reporting high agreement with these items because it might make them seem mean to others.

#### Self-esteem

We measured self-esteem using two scales: a single-item, global trait measure and a six-item, three-dimensional state measure. First, we used the Single-Item Self-Esteem Scale to measures one’s positive or negative feelings about the self at the global trait level [40]. Participants rated their agreement from 1 (s*trongly disagree*) to 9 (s*trongly agree*) with the statement “I have high self-esteem.” Second, we used the Six-Item State Self-Esteem Scale [41] adapted from the 20-item State Self-Esteem Scale [42], which evaluates short-term fluctuations in self-esteem. This measure has three state self-esteem subscales composed of two items each: Social (e.g., “I am worried about what other people think of me” [reverse-scored]), Appearance, (e.g., “I feel satisfied with the way my body looks right now”), and Performance (“I feel frustrated or rattled about my performance”). Participants reported how they felt “right now” and “at this moment” using a response scale of agreement from 1 (*not at all*) to 5 (*extremely*). All three subscales possess good internal consistency and test–retest reliability [41]. Both trait and state self-esteem should correlate negatively with schadenfreude; those who feel poorly about themselves will be more likely to feel happy at another’s mistake.

#### Gratitude

We measured trait gratitude with the Gratitude Adjective Checklist (GAC), a three-item scale that assesses individual differences in grateful emotions in people’s reactions to the positive experiences they have because of another person [43]. The items ask people to rate the amount they felt *grateful*, *thankful*, and *appreciative* “during the past few weeks.” Participants rated their agreement from 1 (*very slightly or not at all*) to 5 (*extremely*). If schadenfreude is primarily rooted in resentment, then it should correlate negatively with gratitude.

### Results

#### Correlations

Zero-order correlations appear below the diagonal in Table 3 The benign and malicious schadenfreude subscales correlated moderately and positively, and showed acceptable reliability. For this study, we considered moderate correlations of |*r*| ≥ .30 (*p*s < .001; [44]) as support for convergent validity. Our schadenfreude scale correlated positively with the six-item schadenfreude measure [11], envy, aggressive humor, and the Dark Triad traits. Aggression also correlated positively with schadenfreude: the BAQ [34,35] subscales of physical aggression, anger, and hostility correlated positively with schadenfreude, but the verbal aggression subscale did not. This null result was surprising because schadenfreude is associated with intentions to spread news of failure [10]. It may be that this is true for episodic—but not trait—schadenfreude, so that only one’s happiness about a *specific* failure will increase the spread of bad news.

**Table 3:** Study 4: Descriptive statistics, zero-order (below diagonal), and partial (above diagonal) correlations (controlling for Krizan & Johar [2012] scale).

Variable	1	2	3	4	5	6	7	8	9	10	11	12	13	14	15	16	17	18	19	20	21	22	23	24	25	26	27	28	29
1. Schadenfreude	—	.84	.76	.20	—	-.32	.01	.52	.24	.33	.15	.05	.04	.43	.08	.30	.38	.11	-.42	-.35	.18	-.22	-.36	-.21	-.24	—	—	—	.00
2. Benign	.85	—	.30	.06	—	-.25	.13	.42	.14	.22	.06	.02	.01	.26	.06	.15	.27	.00	-.26	-.13	.07	-.10	-.27	-.14	-.14	—	—	—	-.01
3. Malicious	.86	.47	—	.30	—	-.28	-.15	.41	.26	.33	.18	.07	.05	.47	.08	.36	.38	.18	-.45	-.46	.24	-.28	-.35	-.20	-.27	—	—	—	.01
4. Krizan & Johar	.62	.40	.67	—																									
5. Envy	.57	.33	.65	.75	—																								
6. Empathy	-.56	-.41	-.55	-.56	-.52	—																							
7. Humor Sense	-.33	-.11	-.45	-.54	-.55	.56	—																						
8. Humor Style	.68	.54	.63	.56	.50	-.52	-.21	—																					
9. DTDD	.57	.37	.61	.71	.66	-.60	-.54	.57	—																				
10. Mach	.61	.41	.63	.66	.60	-.61	-.46	.60	.89	—																			
11. Psych	.50	.31	.54	.66	.62	-.61	-.51	.48	.87	.72	—																		
12. Narc	.33	.20	.36	.47	.45	-.28	-.40	.33	.77	.49	.50	—																	
13. BAQ	.45	.28	.49	.70	.61	-.42	-.49	.47	.72	.62	.68	.53	—																
14. Physical	.43	.27	.47	.56	.42	-.34	-.35	.43	.59	.53	.56	.41	.76	—															
15. Verbal	.08	.06	.08	.21	.14	-.13	-.15	.16	.39	.31	.35	.34	.57	.31	—														
16. Anger	.30	.15	.36	.51	.40	-.35	-.42	.38	.46	.44	.44	.29	.68	.30	.14	—													
17. Hostility	.38	.27	.38	.58	.59	-.31	-.41	.26	.49	.39	.47	.40	.70	.34	.19	.40	—												
18. Extraversion	.11	.00	.18	.13	.06	-.09	-.21	.08	.25	.16	.11	.37	.15	.18	.17	.05	-.00	—											
19. Agreeableness	-.42	-.26	-.45	-.39	-.38	.65	.36	-.38	-.36	-.42	-.41	-.08	-.25	-.22	-.04	-.22	-.20	.12	—										
20. Conscientious	-.35	-.13	-.46	-.35	-.49	.33	.29	-.33	-.37	-.41	-.33	-.17	-.28	-.20	.06	-.36	-.23	-.03	.27	—									
21. Neuroticism	.18	.07	.24	.31	.41	-.14	-.17	.21	.16	.15	.15	.10	.23	.04	-.11	.45	.25	-.19	-.13	-.41	—								
22. Imagination	-.23	-.10	-.28	-.34	-.36	.39	.35	-.14	-.24	-.25	-.30	-.06	-.25	-.13	-.01	-.30	-.25	.17	.40	.24	-.22	—							
23. Desirability	-.36	-.27	-.35	-.39	-.41	.33	.16	-.42	-.40	-.50	-.36	-.14	-.35	-.23	-.01	-.38	-.31	.18	.32	.38	-.39	.22	—						
24. SISE	-.14	-.11	-.13	.03	-.16	.04	-.06	-.07	.13	-.01	.05	.30	.07	.10	.22	-.08	-.06	.30	.16	.27	-.31	.07	.29	—					
25. State SE	-.24	-.14	-.27	-.34	-.47	.16	.14	-.29	-.28	-.28	-.30	-.11	-.28	-.15	.00	-.30	-.30	.27	.26	.44	-.45	.27	.45	.33	—				
26. Social	-.22	-.09	-.29	-.35	-.42	.20	.22	-.29	-.35	-.32	-.33	-.24	-.28	-.19	-.01	-.25	-.29	.15	.27	.31	-.33	.26	.32	.15	.82	—			
27. Appear	.03	-.01	.05	-.03	-.13	-.09	-.16	-.06	.12	.06	.06	.19	-.02	.09	.09	-.15	-.08	.36	.05	.28	-.40	.08	.35	.38	.72	.36	—		
28. Perform	-.36	-.24	-.38	-.42	-.55	.26	.26	-.34	-.40	-.39	-.42	-.19	-.36	-.24	-.06	-.32	-.35	.15	.30	.45	-.36	.30	.41	.27	.87	.62	.44	—	
29. GAC	.00	-.00	.01	.04	.01	.12	-.07	.04	.13	.08	.05	.20	.08	.04	.19	.03	-.02	.23	.21	.06	-.17	.22	.21	.16	.09	.04	.16	.04	—
Descriptive statistics																													
Mean	4.10	4.68	3.53	3.77	2.81	3.49	2.72	3.49	4.78	4.44	4.56	5.33	4.18	4.04	4.65	3.67	4.36	2.87	3.70	3.49	2.81	3.51	1.53	6.28	3.66	3.66	3.60	3.71	3.47
*SD*	1.13	1.31	1.33	1.10	0.78	0.51	0.46	0.91	1.41	1.89	1.54	1.57	0.74	1.30	0.94	1.08	1.00	0.83	0.78	0.74	0.79	0.81	0.20	2.04	0.84	1.05	0.97	1.11	0.72
*Α*	.75	.62	.67	.73	.83	.80	.68	.67	.87	.83	.70	.74	.69	.65	.31	.38	.45	.62	.72	.53	.56	.62	.61	—	.78	.67	.49	.73	.80

Note. *N* = 261 |*r*|s ≥ 0.13, 0.17, and 0.30 have two-tailed *p*s < .05, 01, and .001, respectively

As expected, the six-item alternative schadenfreude measure [11] correlated more positively with trait envy than our schadenfreude scale did. In addition, malicious (vs. benign) schadenfreude correlated more positively with the six-item schadenfreude measure, envy, aggressive humor, the Dark Triad traits, and aggression. The benign subscale did not meet the moderate correlation threshold for substantive support for aggression or Narcissism, but the malicious subscale did.

The Big Five personality traits were related to schadenfreude in both predicted and unexpected ways. As expected, the total schadenfreude scale correlated positively with neuroticism, and negatively with agreeableness and conscientiousness. Whereas the malicious subscale again followed the pattern of the total scale, the benign subscale did not correlate with neuroticism. Although we did not predict it, schadenfreude correlated negatively with openness, and the malicious subscale correlated positively with extraversion. Of the Big Five traits, however, only agreeableness and conscientiousness surpassed the threshold for substantive support, so the other effects should be interpreted with caution. Moreover, for agreeableness and conscientiousness, the benign subscale did not exceed the threshold for substantive support.

Our schadenfreude scale—both as a composite and through its benign and malicious subscales—correlated negatively with empathy, affiliative humor, and social desirability. The effects for *trait* self-esteem did not exceed the threshold for substantive support; however *state* self-esteem in the Performance domain negatively related to both our schadenfreude composite and the malicious—but not benign—subscale. Furthermore, the recurring pattern for the results held for social desirability and affiliative humor, in that the benign subscale did not meet the threshold for substantive support. Gratitude did not correlate with any schadenfreude measure.

#### Incremental validity

Table 3 above the diagonal, shows partial correlations controlling for the six-item schadenfreude measure [11] for our schadenfreude scale and its benign and malicious subscales. As expected, controlling for a related measure weakened some correlations; however, the general pattern of correlations across the nomological network remained. We also ran multiple regressions where we entered the six-item schadenfreude at Step 1 and our benign and malicious schadenfreude subscales in Step 2. We assessed incremental validity by examining change statistics between Steps 1 and 2 (Table 4). As a set, benign and malicious schadenfreude showed evidence of incremental validity over the six-item schadenfreude measure in 13 of 22 (59%) outcomes assessed.

**Table 4:** Incremental validity statistics for benign and malevolent schadenfreude, controlling for Krizan and Johar’s scale [11].

	Change statistics			Partial correlations^a^	
Outcome measure	Δ*R*^2^	Δ*F*_2, 257_	*p* ≤	Benign	Malevolent
Envy	.039	12.72	.001	-.04	.30*
Empathy	.079	15.49	.001	-.18*	-.22*
Humor Sense	.038	7.20	.001	.18*	-.19*
Humor Style	.185	47.15	.001	.34*	.33*
DTDD mean	.036	10.11	.001	.06	.24*
Machiavellianism	.072	18.72	.001	.13*	.29*
Psychopathy	.019	4.47	.012	.01	.17*
Narcissism	.004	0.66	.517	-.00	.07
BAQ mean	.001	0.37	.689	-.01	.05
Physical Aggression	.018	3.34	.037	.01	.15*
Verbal Aggression	.006	0.85	.429	-.01	-.07
Anger	.005	0.86	.425	-.08	.05
Hostility	.003	0.50	.608	.06	-.03
Extraversion	.025	3.33	.037	-.10	.15*
Agreeableness	.065	10.76	.001	-.05	-.25*
Conscientiousness	.108	18.04	.001	.12*	-.35*
Neuroticism	.007	1.06	.347	-.08	.07
Imagination	.010	1.44	.238	.07	-.10
Social Desirability	.024	3.76	.024	-.11	-.09
Single-Item Self-Esteem	.047	6.39	.002	-.08	-.17*
State Self-Esteem	.003	0.45	.639	.01	-.06
Gratitude	.001	0.10	.909	-.02	-.01

Note. DTDD = Dark Triad Dirty Dozen [32]. BAQ = Brief Aggression Questionnaire [34].

^a^Partial correlations from a three-predictor model: (1) Krizan and Johar’s Scale [11], (2) Benign Schadenfreude, and (3) Malevolent Schadenfreude.

*p < .05.

### Discussion

Our 12-item schadenfreude scale correlated with other scales as predicted and showed evidence of incremental validity over another schadenfreude measure. Our measure showed a reasonable pattern of relationships for the construct of schadenfreude, though it appeared that the malicious subscale had stronger relationships with our chosen measures than its benign counterpart. That gratitude failed to correlate with schadenfreude is unsurprising, because while resentment may contribute to episodic schadenfreude, resentment is not necessary for schadenfreude to occur; resentment and schadenfreude may simply be unrelated at the trait level. What remains unclear is whether our schadenfreude measure relates to *episodic* schadenfreude, such that being higher in *trait* schadenfreude generally relates to reporting higher schadenfreude in response to a vignette.

## Study 5: Schadenfreude and spreading news of political failure

Because schadenfreude may be a natural consequence of an outgroup’s misfortune [7], we gave participants a fictional news story that described a politician’s downfall in Study 5. The politician was either from the same party as the participant, or the opposing party. A strength of this design is that participants were led to believe this was a real event, unlike other research on schadenfreude that presents vignettes as hypotheticals. One exception [45] found that Australian participants reacted with pleasure to the real failures of real politicians from an opposing party. However, our study benefits from using new, ostensibly real politicians to whom participant had no preconceived attitudes. We expected that participants’ reported feelings of schadenfreude—both overall and in reaction to specific stories—should predict their choices to share those stories with others via social media, particularly for the outgroup’s failures (people who self-identify as Republican vs. Democrat). Participants provided both a measure of *intent* to share the story and a *behavioral* measure of the same: they were given the opportunity to ostensibly share the information via their Facebook or Twitter accounts. We also included a non-politically-partisan control condition in which participants read about a similar gaff from chief executive officer (CEO).

### Method

#### Participants

Participants were 355 MTurk members from the U.S. who we compensated US51¢. We included three attention-check questions throughout the survey, and excluded participants who failed one or more. We also excluded participants who did not know who Robert Hunter was after following the manipulation (see below). As a result, we retained 304 participants (86%) for analysis (118 men, 184 women, 2 unspecified; ages: 18–75 years, *M =* 39.7, *SD =* 14.4). There were 120 Democrats, 72 Republicans, and 112 people who listed another party affiliation.

#### Materials and procedure

The study was a 3 (Affiliation of participant: Democrat, Republican, or other) × 3 (Condition: Democrat, Republican, or CEO) design. First, participants reported the political party with whom they affiliated and the extent to which they identified with their party. For affiliation, participants responded to “If you are a U.S. citizen or resident, which of the following political parties do you identify with most?” The options were *Democrat*, *Green Party*, *Libertarian*, *Republican*, *other/no party affiliation*, and *not a U*.*S*. *citizen or resident*. For identification, participants responded to “How much do you identify with your political party? (If you don’t affiliate with either, select *not at all*)” using a scale from 1 (*not at all*) to 5 (*extremely*). Participants completed various personality measures, including our 12-item trait schadenfreude scale (see Studies 2–4) and other measures (e.g., dispositional envy; [12]) to disguise the purpose of the experiment. Participants also completed a short-form social desirability scale [37–39]. The response scales for these measures were the same as in Study 4.

Participants completed an altered version of the 16-item Collective Self-Esteem Scale [46] designed for this study as a measure of investment in one’s political party. Theory underlying the Collective Self-Esteem Scale argues that one’s collective social identity is important to understanding group-level behaviors (e.g., in-group bias). There are four 4-item Collective Self-Esteem subscales: Membership (e.g., “I am a worthy member of the social groups I belong to”), Private (e.g., “I feel good about the social groups I belong to”), Public (e.g., “Overall, my social groups are considered good by others”), and Identity (e.g., “The social groups I belong to are an important reflection of who I am”). While the original scale is for one’s generic social group, there are versions specifically designed to assess racial identity [47] and family-based collective self-esteem [48]. For Study 5, we altered all 16 items to reflect *political* affiliation (e.g., “The political party I belong to is an important reflection of who I am”). Participants rated their agreement from 1 (s*trongly disagree*) to 7 (s*trongly agree*).

Some measures were included as a test of the mechanism of schadenfreude. For example, it may be that schadenfreude is simply a way to raise self-esteem (Six-Item State Self-Esteem Scale; [41]), or perhaps a result of a general tendency toward downward social comparison (Self-Attributes Questionnaire; SAQ; [49]). The SAQ is a 10-item social comparison measure with good internal consistency. Participants rated themselves relative to other people their own age. The SAQ used a ten-point scale from A (bottom 5%) to J (top 5%). Finally, we included an alternative six-item schadenfreude measure [11] for comparison with our new 12-item schadenfreude scale.

Participants read one of 18 vignettes (S1 Appendix), presented as a news article (S2 Appendix), about a prominent person’s downfall because of financial or sexual scandals. For generalizability and stimulus sampling, we created six variations on the vignettes, which we then altered to focus on a Democrat, a Republican, or a CEO [50,51]. This meant that the reaction to the vignettes were not based on idiosyncratic features of any one stimulus, but were instead based on a set of stimuli reflecting party and type of scandal. Participants reported how they felt (episodic schadenfreude) by responding to two similar statements: “How [pleased/‘secretly happy’] were you when you found out Robert Hunter would likely be facing legal action.” Both items were averaged together to create the episodic schadenfreude variable (*r* = .66, α = .79). Participants also reported their intentions to share the story on a scale from 1 (*Very Unlikely*) to 7 (*Very Likely*), and whether they would like to share the story “automatically” on Facebook or Twitter. Participants had four response choices: no, no because they did not have an account at the site, yes, and yes but he or she would do it later. Because of social media’s popularity, one’s decision to share news with their friends and followers can be considered a valid measure of communication behavior. Finally, we informed participants about the fictitious nature of the study and debriefed them.

### Results and discussion

**Fig 4:**
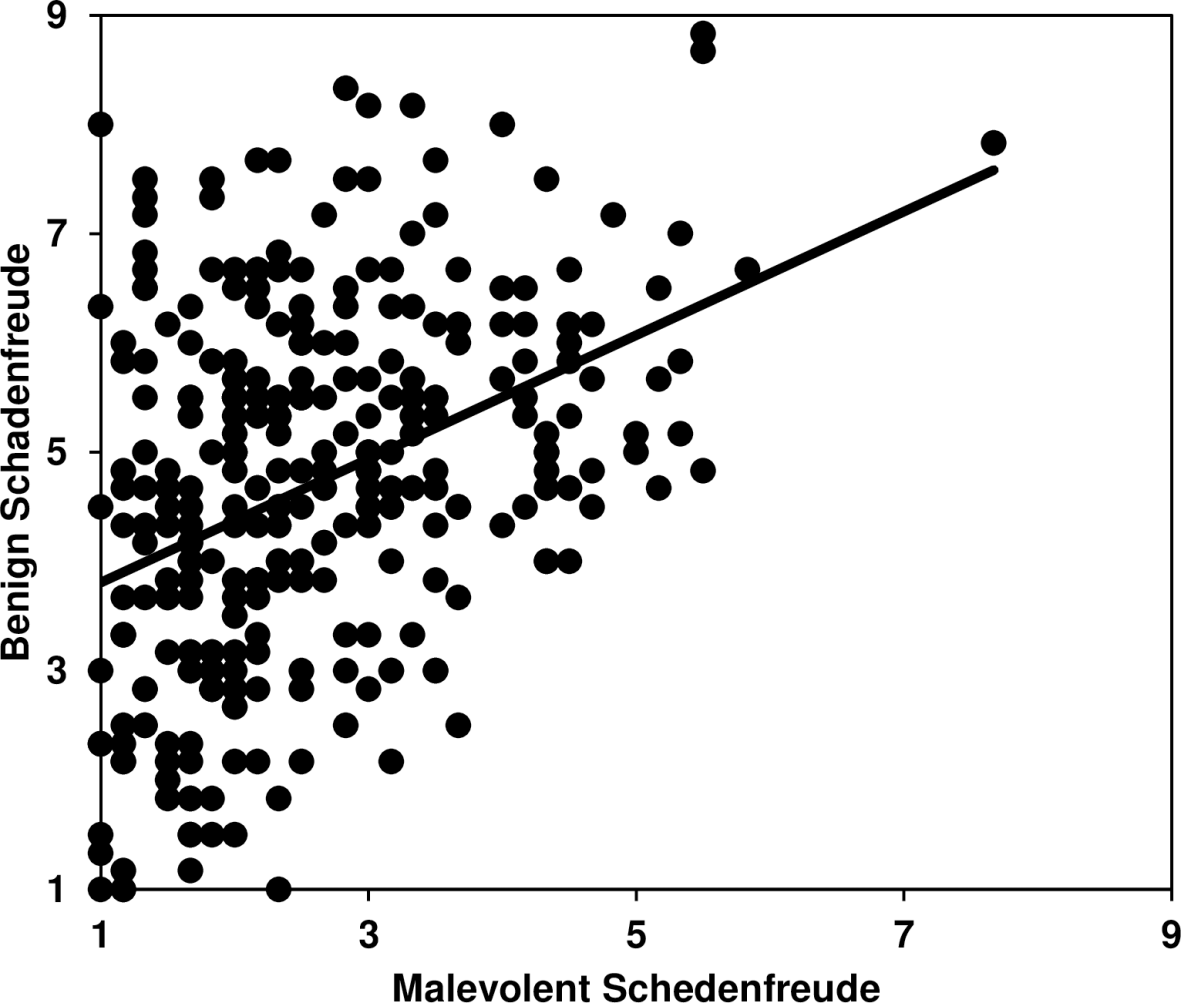
Subscale scatterplot. Study 5: Scatterplot showing the distributions of the Malevolent (*x*-axis) and Benign (*y*-axis) Schadenfreude subscales. *N* = 304, *r* = .39, *p* < .001.

Reliability was good for the total schadenfreude measure (*α =* .79, *M* = 3.65, *SD* = 1.17) and its benign (*α =* .77, *M* = 4.71, *SD* = 1.65) and malicious (*α =* .73, *M* = 2.59, *SD* = 1.14) subscales, which were positively correlated (*r* = .39, *p* < .001; Fig 4).

**Fig 5:**
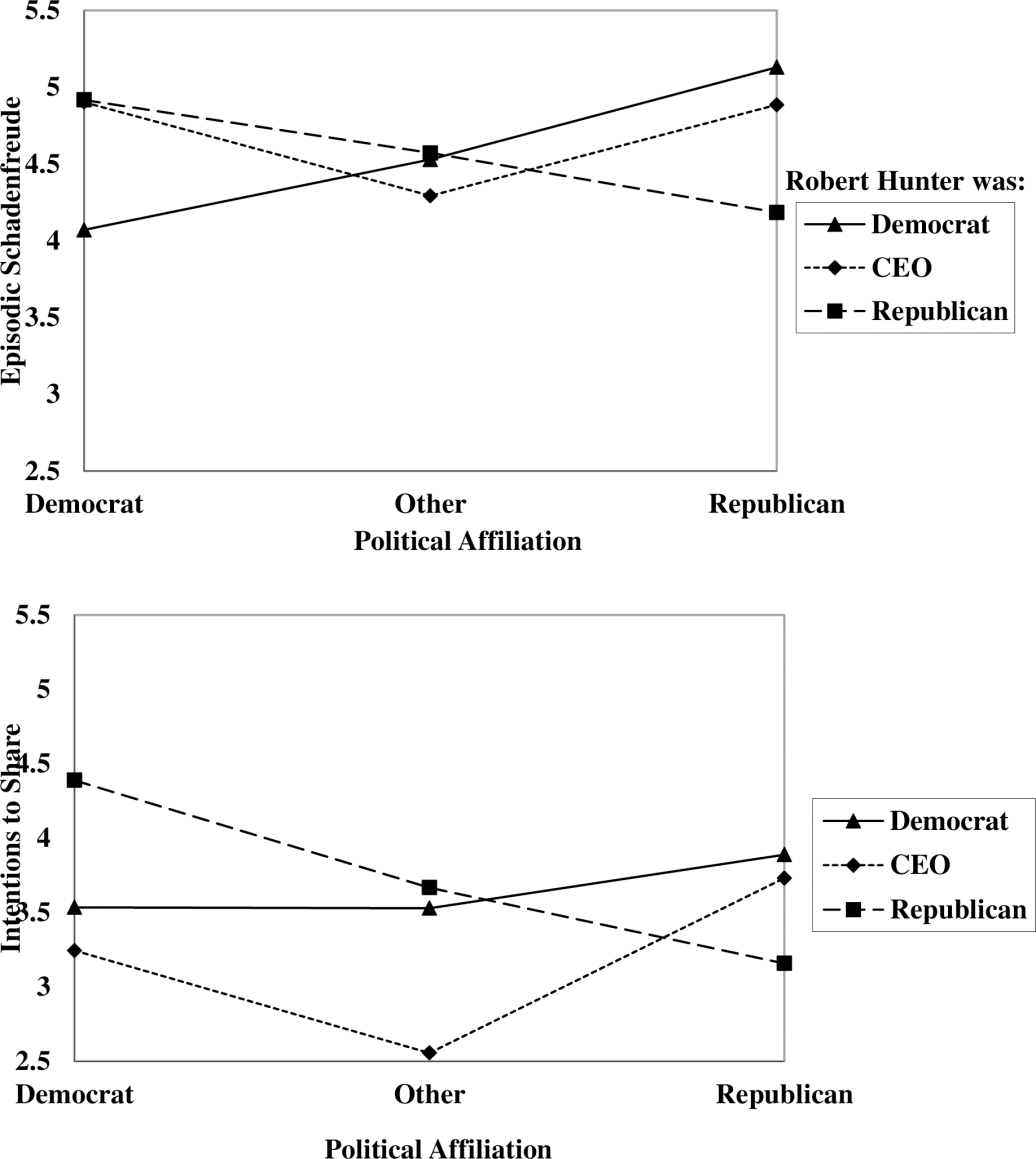
Decomposed interactions. Top: Episodic schadenfreude as a function of condition and political affiliation. Bottom: Intentions to share as a function of condition and political affiliation. Study 5: Focal effects from the bootstrapped moderated mediation model described in Fig 6. Numbers reflect unstandardized regression coefficients, with 95% confidence intervals in brackets. The unmediated direct effect is shown in parentheses.

Given our predictions, we tested the focused, single-degree-of-freedom contrast codes that specifically compared the 2 (Affiliation: Democrat [-0.5] vs. Republican [0.5]; Other [0.0]) × 2 (Condition: Democrat [-0.5] vs. Republican [0.5]; CEO [0.0]) interaction within the broader 3 × 3 design ([52]; see Table 5‘s boldfaced effect). This 2 (Affiliation) × 2 (Condition) interaction showed that the manipulation had the expected effect: episodic schadenfreude was higher when the downfall occurred for someone from an opposing political party than for someone from one’s own political party (*b* = -1.79, *t*_295_ = -3.96, *p* < .001, *d* = -0.46; Fig 5, top).

**Table 5:** Episodic schadenfreude (left) and intentions to share robert hunter’s story (right) as functions of affiliation and condition.

	Episodic Schadenfreude				Intentions to Share			
Variable	*b*	*t*_295_	*p ≤*	*r*_p_	*b*	*t*_295_	*p ≤*	*r*_p_
Intercept	4.61	64.35	.001	—	3.52	34.76	.001	—
Affiliation contrast 1: Democrat vs. Republican	0.10	0.57	.570	.03	-0.13	-0.51	.612	-.03
Affiliation contrast 2: Partisan vs. Other	0.22	1.48	.139	.08	0.41	1.97	.050	.11
Condition contrast 1: Democrat vs. Republican	-0.02	-0.11	.915	-.01	0.09	0.35	.730	.02
Condition contrast 2: Politician vs. CEO	-0.13	-0.84	.401	-.05	0.52	2.44	.015	.14
**Affiliation contrast 1** × **Condition contrast 1**	**-1.79**	**-3.96**	**.001**	**-.22**	**-1.59**	**-2.47**	**.014**	**-.14**
Affiliation contrast 1 × Condition contrast 2	0.18	0.48	.632	.03	-0.93	-1.73	.085	-.10
Affiliation contrast 2 × Condition contrast 1	-0.09	-0.25	.800	-.02	-0.08	-0.15	.882	-.01
Affiliation contrast 2 × Condition contrast 2	-0.58	-1.87	.063	-.11	-0.79	-1.80	.073	-.10

Note. **Boldface** = predicted 2 (Affiliation: Democrat vs. Republican) × 2 (Condition: Democrat vs. Republican) interaction.

We next decomposed this 2 (Affiliation) × 2 (Condition) interaction by examining the four possible simple differences using contrast codes to make focused comparisons using established procedures [52–54]. Any 2 × 2 interaction can be broken down into four simple slopes or effects or differences. For example, imagine a 2 (Sex: men vs. women) × 2 (Manipulation: experiment vs. control) design. One could examine the simple sex difference between the men and women among those in (a) the experimental condition and (b) the control condition; and one could examine the simple manipulation difference (i.e., experiment vs. control) among (c) men and (d) women. Thus, any 2 × 2 interaction can yield four simple effects or difference tests that present unique pieces of information.

First, people affiliating as Democrats reported higher schadenfreude in the Robert Hunter–Republican condition (*M* = 4.92) than the Robert Hunter–Democrat condition (*M* = 4.07), respectively (*b* = 0.85, *t*_295_ = 3.10, *p* = .002, *d =* 0.36; Fig 5, top, cf. leftmost square and triangle). Second, people affiliating as Republicans reported higher schadenfreude in the Robert Hunter–Democrat condition (*M* = 5.13) than in the Robert Hunter–Republican condition (*M* = 4.18), respectively (*b* = -0.94, *t*_295_ = -2.61, *p* = .009, *d* = -0.30; Fig 5, top, cf. rightmost square and triangle). Third, people assigned to the Robert Hunter–Democrat condition reported higher schadenfreude if they affiliated as Republicans (*M* = 5.15) than as Democrats (*M* = 4.07; *b* = 1.06, *t*_295_ = 3.57, *p* < .001, *d* = 0.42; Fig 5, top, cf. rightmost and leftmost triangles at the ends of the solid line). Fourth, people assigned to the Robert Hunter–Republican condition reported higher schadenfreude if they affiliated as Democrats (*M* = 4.92) than as Republicans (*M* = 4.18; *b* = -0.73, *t*_295_ = -2.14, *p* = .03, *d* = -0.25; Fig 5, top, cf. rightmost and leftmost squares at the ends of the dashed line).

Trait schadenfreude, as measured by our scale, predicted episodic schadenfreude even after controlling for the experimental manipulation (*b* = 0.13, *t*_294_ = 2.17, *p* = .03, *r*_p_ = .13), but this was only marginally true when its benign (*b* = 0.081, *t*_294_ = 1.90, *p* = .06, *r*_p_ = .11) or malicious (*b* = 0.10, *t*_294_ = 1.69, *p* = .09, *r*_p_ = .10) subscales were used. Showing some convergent validity, the six-item schadenfreude measure [11] also positively related to episodic schadenfreude (*b* = 0.19, *t*_294_ = 3.06, *p* = .002, *r*_p_ = .18). Neither benign (*b* = 0.048, *t*_292_ = 1.04, *p* = .30, *r*_p_ = .061) nor malicious (*b* = -0.080, *t*_292_ = -0.90, *p* = .37, *r*_p_ = -.053) schadenfreude related to episodic schadenfreude after controlling for the experimental manipulation and the six-item schadenfreude measure. We suspect that this lack of incremental validity (Δ*R*^2^ = .005, Δ*F*_2, 292_ = 0.79, *p* = .45) may be because episodic and trait schadenfreude reflect (a) conceptually different time frames similar to distinguishing state from trait assessments or (b) different levels of specificity given that the episodic schadenfreude measure pertained specifically to the experiment manipulation (i.e., reactions to Robert Hunter), whereas our trait schadenfreude measure reflects people’s dispositional tendencies. To be sure, state or episodic measures should generally correlate positively with the respective trait measures because traits can be an expression of an aggregation of people’s states over a variety of situations [55]. Nevertheless, although lack of incremental validity is problematic, we reiterate that our new trait schadenfreude scale did indeed show convergent validity with the episodic schadenfreude items above and beyond the effects of a successful experimental manipulation.

Our schadenfreude scale did not relate to intentions to share the story with others (*r =* .033), but episodic schadenfreude did (*r =* .27, *p <* .001). We ran a multiple regression (Table 5), and again found support for our planned 2 (Affiliation) × 2 (Condition) interaction (within the 3 × 3 design) for intentions to share, which were higher when the downfall occurred for someone from the opposing (vs. same) political party (*b* = -1.59, *t*_295_ = -2.47, *p* = .01, *d* = -0.29; Fig 5, bottom). We decomposed this interaction by examining the four possible simple by using contrast codes to make focused comparisons [53]. First, people affiliating as Democrats reported higher intentions to share in the Robert Hunter–Republican condition (*M* = 4.39) than the Robert Hunter–Democrat condition (*M* = 3.54), respectively (*b* = 0.85, *t*_295_ = 2.21, *p* = .03, *d =* 0.26). In contrast, people affiliating as Republicans did not report significantly higher intentions to share in the Robert Hunter–Democrat condition (*M* = 3.89) than in the Robert Hunter–Republican condition (*M* = 3.16), respectively (*b* = -0.73, *t*_295_ = -1.43, *p* = .15, *d* = -0.17). Third, people assigned to the Robert Hunter–Democrat condition did not report higher intentions to share if they affiliated as Republicans (*M* = 3.89) than as Democrats (*M* = 3.54; *b* = 0.35, *t*_295_ = 0.84, *p* = .40, *d* = 0.10). Fourth, people assigned to the Robert Hunter–Republican condition reported higher schadenfreude if they affiliated as Democrats (*M* = 4.39) than as Republicans (*M* = 3.16; *b* = -1.23, *t*_295_ = -2.54, *p* = .01, *d* = -0.30).

Episodic schadenfreude—but not trait schadenfreude—predicted intentions to share the story even after controlling for the Affiliation × Condition interaction (*b* = 0.36, *t*_294_ = 4.47, *p* < .001, *d* = 0.52, *r*_p_ = .25), and the Affiliation × Condition interaction of interest was no longer significant after accounting for episodic schadenfreude (*b* = -0.94, *t*_294_ = -1.48, *p* = .14, *d* = -0.17). Neither benign (*b* = -0.029, *t*_292_ = -0.43, *p* = .66, *r*_p_ = -.025) nor malicious (*b* = -0.020, *t*_292_ = -0.16, *p* = .88, *r*_p_ = -.009) Schadenfreude related to intensions to share after controlling for the experimental manipulation and the six-item schadenfreude measure. This lack of incremental validity (Δ*R*^2^ = .001, Δ*F*_2, 292_ = 0.12, *p* = .88) was unsurprising because not even the six-item schadenfreude measure significantly related to intensions to share the story (*r*_p_ = .073, *p* = .21).

Neither episodic nor trait schadenfreude predicted agreeing to share the story on Facebook or Twitter. This is likely because only 31 of 276 participants (11%) responded “share” to this question; most participants were simply not interested in posting the story on Facebook or Twitter. Moreover, state self-esteem, the SAQ, and collective self-esteem were unrelated to our schadenfreude scale. Nevertheless, people that scored higher on the Political Affiliation Collective Self-esteem subscales for Membership (*r* = .15, *p* = .01) and Identity (*r* = .12, *p* = .03) reported higher *intentions* of sharing the story with others; they were not, however, more likely to share the story on Facebook or Twitter.

**Fig 6:**
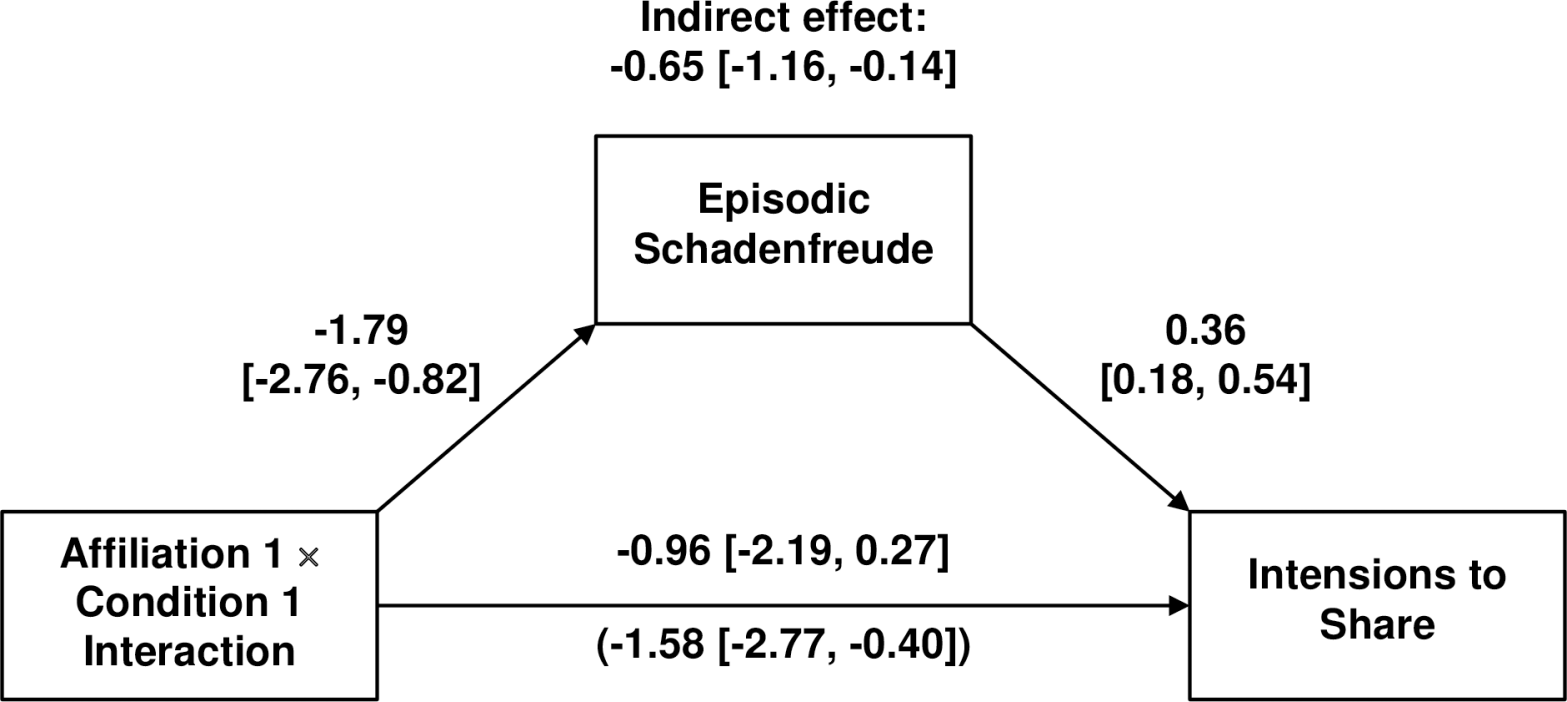
Bootstrapped moderated mediation model.

As an exploratory exercise, we examined mediated moderation following established guidelines [56] and tested the indirect effect using bootstrapped standard errors (10,000 draws; [57]; Table 6; Fig 6). Whereas statistical moderation is another term for an interaction effect, mediated moderation occurs when the direct effect of an interaction effect on an outcome is significantly reduced in magnitude after adding another predictor—the purported mediator—to the regression model. Mediated moderation is present if the “indirect effect” of the interaction on the outcome via the mediator is significant, or equivalently, the direct effect of the interaction on the outcome is significantly reduce after adding the mediator. Episodic schadenfreude fully and significantly mediated the predicted affiliation by condition interaction (a) because the direct effect of the predicted interaction on intensions to share was reduced to non-significance after controlling for episodic schadenfreude (cf. Tables 5 & 6) and (b) because the indirect effect through episodic schadenfreude was significant (indirect effect = -0.65, *z* = -2.50, *p* = .012).

**Table 6:** Episodic schadenfreude mediates the predicted affiliation × condition contrast.

Variable	*b*	*t*	*p ≤*	*r*_p_
Episodic Schadenfreude (ES; mean-centered)				
Intercept	0.01	0.13	.900	—
Affiliation contrast 1: Democrat vs. Republican	0.10	0.54	.589	.03
Affiliation contrast 2: Partisan vs. Other	0.22	1.52	.128	.09
Condition contrast 1: Democrat vs. Republican	-0.02	-0.10	.922	-.01
Condition contrast 2: Politician vs. CEO	-0.13	-0.87	.383	-.05
**Affiliation contrast 1 × Condition contrast 1**	**-1.79**	**-3.63**	**.001**	**-.21**
Affiliation contrast 1 **×** Condition contrast 2	0.18	0.47	.636	.03
Affiliation contrast 2 **×** Condition contrast 1	-0.09	-0.25	.801	-.01
Affiliation contrast 2 **×** Condition contrast 2	-0.58	-2.02	.044	-.12
Intentions to Share				
Intercept	3.54	35.22	.001	—
Affiliation contrast 1: Democrat vs. Republican	-0.12	-0.45	.656	-.03
Affiliation contrast 2: Partisan vs. Other	0.33	1.58	.114	.09
Condition contrast 1: Democrat vs. Republican	0.05	0.18	.855	.01
Condition contrast 2: Politician vs. CEO	0.58	2.72	.006	.16
**Affiliation contrast 1 × Condition contrast 1**	**-0.96**	**-1.53**	**.126**	**-.09**
Affiliation contrast 1 **×** Condition contrast 2	-0.90	-1.67	.095	-.10
Affiliation contrast 2 **×** Condition contrast 1	-0.09	-0.18	.860	-.01
Affiliation contrast 2 **×** Condition contrast 2	-0.44	-1.00	.316	-.06
Episodic Schadenfreude (ES; mean-centered)	0.36	3.91	.001	.23
ES **×** Affiliation contrast 1: Democrat vs. Republican	-0.06	-0.27	.790	-.02
ES **×** Affiliation contrast 2: Partisan vs. Other	-0.05	-0.21	.831	-.01
ES **×** Condition contrast 1: Democrat vs. Republican	0.07	0.36	.717	.02
ES **×** Condition contrast 2: Politician vs. CEO	-0.20	-0.97	.332	-.06
ES **×** Affiliation contrast 1 **×** Condition contrast 1	0.34	0.65	.512	.04
ES **×** Affiliation contrast 1 **×** Condition contrast 2	-0.18	-0.37	.714	-.02
ES **×** Affiliation contrast 2 **×** Condition contrast 1	0.67	1.33	.184	.08
ES **×** Affiliation contrast 2 **×** Condition contrast 2	0.38	0.92	.356	.05

Note. **Boldface** = predicted 2 (Affiliation: Democrat vs. Republican) **×** 2 (Condition: Democrat

## General discussion

Schadenfreude is a social emotion involving joy at the downfall of others. The present research suggests that people also show substantial individual differences in schadenfreude. We developed a 12-item, two-factor schadenfreude measure. Regarding incremental validity, our two-factor schadenfreude scale was able to explain a significant amount of variance over and above Krizan and Johar’s one-factor schadenfreude scale [11] in nearly 60% of the outcomes examined in Study 4. Establishing incremental validity in Study 5 was more challenging because (a) *episodic* schadenfreude differs considerably from our measure’s focus on *trait* schadenfreude and (b) neither our measure nor Krizan and Johar’s [11] related to willingness to share a story about a political downfall. Also recall that Krizan and Johar’s [11] scale was ad-hoc measure of episodic schadenfreude developed for a study focusing on narcissism; consequently, it did not undergo rigorous psychometric testing. Thus, the two-dimensional trait nature of our scale provides a broader, more comprehensive measure of the schadenfreude construct in most cases. Because of its broader, two-factor approach, and the incremental validity it showed over a prior measure [11], we believe our schadenfreude scale provides more valid and reliably scores than earlier measures.

Schadenfreude is rarely studied as a personality trait. If schadenfreude is part of a growing emphasis on “dark” personality traits (cf. The Dark Triad; [29,58]), then having a psychometrically validated trait measure of it should help spur further research on schadenfreude and its causes, correlates, and consequences. Models of personality traits—and how they influence our political psychology—may be incomplete without attention to the “darker” aspects of humanity.

### Implications, limitations, and future directions

The main implications of the present research are twofold. First, we have developed the first multidimensional and psychometrically validated schadenfreude scale. We believe that the benign and malicious subscales can be used in conjunction to gain a better understanding of the dynamic nature of individual differences in trait schadenfreude. Importantly, this research highlights that some aspects of trait schadenfreude are fairly harmless (benign), whereas others are inherently harmful (malicious). We hope that this augmented conceptualization of schadenfreude will help spur further research into why some people express more schadenfreude than others. Moreover, controlling for individual differences in schadenfreude may make it easier to measure the situational antecedents or behavioral consequences of schadenfreude. Researchers interested in “dark” personality traits including schadenfreude can use our individual difference measure to examine person-by-situation interactions. Our scale’s two-factor approach also allows for the possibility of dynamic or interactive associations between benign and malicious schadenfreude. For example, future researcher may wish to test whether people with the specific combination of low benign and high malicious schadenfreude show the most behavioral aggression following an insult [48].

Second, the present research has clear implications for political psychology with respect to episodic schadenfreude and willingness to share embarrassing stories about public figures on social media. Specifically, (a) one’s experience of episodic schadenfreude appears to depend on one’s political affiliation and whether the target is of the same or different affiliation, and (b) episodic schadenfreude played a key role in mediating the crucial Affiliation-by-Condition interaction in our Study 5 experiment predicting one’s willingness to share stories of political downfall on social media. Thus, in two-party political environments in which one party’s scandal inherently benefits the other party, schadenfreude is likely a substantial driving force worthy of further research by social, personality, media, and political psychologists.

Despite extensive psychometric testing across five studies, our 12-item schadenfreude scale has limitations. In Study 3, the malicious subscale showed poor internal reliability at one time point; however, it faired far better at another time point and in other, larger samples. Given psychology’s recent emphasis on behavioral measures [59], another weakness of this research is its reliance on self-reports. Our behavioral measure (social media sharing in Study 5) was unrelated to most self-report measures. Although self-report measures have multiple biases [22], we likely reduced social desirability and positive response bias by reverse-scoring half the items in our 12-item schadenfreude scale.

This choice, however, has both potential benefits and drawbacks. Having reverse-scored items can reduce acquiescence bias in self-reports scales because people tend to agree with statements more than disagree with them [22]. Such items can also help identify inattentive respondents because attentive respondent are unlikely to have extreme scores in the same direction on reverse-scored items. Nevertheless, scales with reverse-scored items often have lower reliability, and there are multiple scoring options. We believe many researchers will simply opt to reverse-score the relevant six items and calculate means across both the benign and malicious subscale items. While this is acceptable, those means may contain some noise related to method variance. A more optimal scoring technique involves estimating the three-factor model shown in Fig 3 and calculating subscale means weighted by the factor scores for benign and malicious schadenfreude; this should reduce unwanted method variance.

Another possible limitation is that we only assessed general envy (vs. benign and malicious envy), and thus do not know how our schadenfreude subscales relate to different forms of envy. Research conducted after our data collection ended suggests that malicious—but not benign—envy relates to schadenfreude [60,61]. Moreover, benign and malicious envy are not perfect parallels of benign and malicious schadenfreude these respective scales assess them. For example, benign envy focuses on the desired object, due to wanting to improve one’s own situation, whereas malicious envy focuses on the envied person, due to wanting to bring them down. Further research will be needed to establish the finer similarities and differences among these forms of envy and schadenfreude.

Schadenfreude may be especially relevant to politics if it strengthens group affiliation. If a person high in trait schadenfreude is more likely to seek out and share stories of politicians’ gaffs, scandals, and misfortunes, then this may interact with party affiliation to decrease faith in the political system, but especially for the opposing party members. For example, if Democrats are spreading the news of a Republican’s failure, then they will likely spread it to other Democrats through both selective exposure to liberal media and selective sharing to likeminded online friends and followers [62]. Certainly the events of the 2016 U.S. Presidential election confirmed that both parties use the scandals of the opposing candidate as confirmation of their prior beliefs. When schadenfreude meets social media, a campaign could be lost overnight.

### Conclusions

Schadenfreude varies not only across scenarios, but also among persons. Some people are higher in schadenfreude than others, being more likely to feel pleasure at others’ misfortunes. We developed a scale in five studies to measure this trait and tested its relationship with other, related measures. We also examined how schadenfreude relates to political affiliation and a politician’s downfall. As U.S. politics become more divisive, and social media are used to shame and denigrate targeted politicians, schadenfreude can increasingly explain our social behavior. If schadenfreude explains why people share these embarrassing failures, then it may even help explain political outcomes, including election victories.

## Supporting information

S1 Appendix Text of scandal stimuli. (DOCX)

S2 Appendix Sample scandal stimuli (as seen by participants). (DOCX)
