# Supplementary material for: Schadenfreude and the spread of political misfortune
Source: PLoS One. 2018 Sep 5;13(9):e0201754. doi: 10.1371/journal.pone.0201754 (PMC6124730; doi:10.1371/journal.pone.0201754)
Supplement: S1 Appendix — (DOCX) [file pone.0201754.s001.docx]

| **S1 Appendix. Text of scandal stimuli.** |
| --- |
| 1. Robert Hunter, (Democrat and member of the House Ethics Committee/ Republican and member of the House Ethics Committee/prominent CEO of a private drafting company) is having a very bad day. The multi-millionaire, who inherited his sizable fortune, has lost at least half of his fortune in a Ponzi scheme for which he was a founding member. In an attempt to illegally increase his already substantial wealth, Mr. Hunter would have benefitted from the loss of hundreds of middle-class Americans. Instead, his pockets are considerably lighter and he will likely be facing criminal charges. |
| 2. Robert Hunter, (Democrat and member of the House Ethics Committee/ Republican and member of the House Ethics Committee/prominent CEO of a private drafting company) is having a very bad day. The multi-millionaire, who inherited his sizable fortune, was caught on tape attempting to solicit the votes of key members of Congress through bribery. In an attempt to illegally increase his already substantial wealth, Mr. Hunter would have benefitted directly from taxpayers dollars. Instead, his pockets are considerably lighter and he will likely be facing criminal charges. |
| 3. Robert Hunter, (Democrat and member of the House Ethics Committee/ Republican and member of the House Ethics Committee/prominent CEO of a private drafting company) is having a very bad day. The multi-millionaire, who inherited his sizable fortune, most certainly used his father’s business ties to defraud the American people. In an attempt to illegally increase his already substantial wealth, Mr. Hunter would have benefitted from the loss of Americans of all financial backgrounds. Instead, his pockets are considerably lighter and he will likely be facing criminal charges. |
| 4. Robert Hunter, (Democrat and member of the House Ethics Committee/ Republican and member of the House Ethics Committee/prominent CEO of a private drafting company) is having a very bad day. Famous for calling his opponents “sexually retarded,” Mr. Hunter has been caught in a sex scandal that will most certainly make him eat his words. Photos of the married multi-millionaire, who inherited his sizable fortune, have been verified to show him being confronted by no less than three mistresses. He will most likely be looking for a new job in the near future, and may be facing legal action for a corruption case related to one of the ladies in question and her involvement in his professional history. |
| 5. Robert Hunter, (Democrat and member of the House Ethics Committee/ Republican and member of the House Ethics Committee/prominent CEO of a private drafting company) is having a very bad day. Famous for calling his opponents “sexually retarded,” Mr. Hunter has been caught in a sex scandal that will most certainly make him eat his words. The married multi-millionaire, who inherited his sizable fortune, has been carrying on an online affair without knowing the true identity of his paramour. The “lady” in question was actually a man engaging in an elaborate hoax. He will most likely be looking for a new job in the near future and may be facing legal action. |
| 6. Robert Hunter, (Democrat and member of the House Ethics Committee/ Republican and member of the House Ethics Committee/prominent CEO of a private drafting company) is having a very bad day. Famous for calling his opponents “sexually retarded,” Mr. Hunter has been caught in a sex scandal that will most certainly make him eat his words. The multi-millionaire, who inherited his sizable fortune, most certainly engaged in sexual relations with a minor. Famous for accusing two of his opponents of similar acts, it appears that he was actually the guilty party. He will most likely be looking for a new job in the near future, and may be facing legal action. |
