# Supplementary figures and images for: Schadenfreude and the spread of political misfortune
Source: PLoS One. 2018 Sep 5;13(9):e0201754. doi: 10.1371/journal.pone.0201754 (PMC6124730; doi:10.1371/journal.pone.0201754)

**S2 Appendix. Sample scandal stimuli (as seen by participants*).***


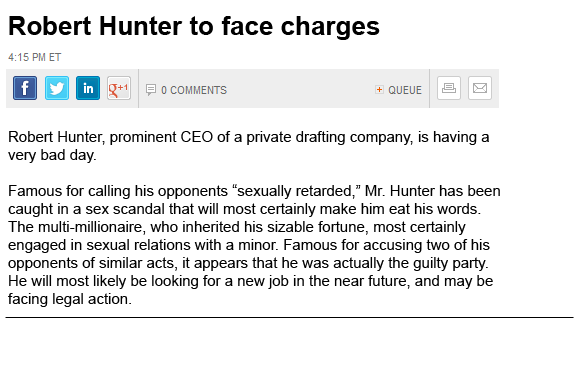

Supplement: S2 Appendix — (DOCX) [file pone.0201754.s002.docx]
